# Energetic-protein supplementation in the last 60 days of gestation improves performance of beef cows grazing tropical pastures

**DOI:** 10.1186/s40104-017-0209-x

**Published:** 2017-10-01

**Authors:** Aline Gomes da Silva, Mário Fonseca Paulino, Edenio Detmann, Henrique Jorge Fernandes, Lincoln da Silva Amorim, Román Enrique Maza Ortega, Victor Valério de Carvalho, Josilaine Aparecida da Costa Lima, Felipe Henrique de Moura, Mariana Benevides Monteiro, Jéssika Almeida Bitencourt

**Affiliations:** 1 Universidade Federal de Viçosa, Viçosa, Minas Gerais 36570–000 Brazil; 2 Present Address: Universidade Federal de Mato Grosso do Sul, Campo Grande, Mato Grosso, do Sul 79074–460 Brazil; 3 Universidade Estadual de Mato Grosso do Sul, Aquidauana, Mato Grosso, do Sul 79200–000 Brazil; 4 Biotran - Biotecnologia e Treinamento em Reprodução Animal, Alfenas, Minas Gerais 37130–000 Brazil; 5 Universidade Federal do Acre, Rio Branco, Acre 69920–900 Brazil

**Keywords:** Flushing, Nutrition, Parturition, Reproduction

## Abstract

**Background:**

Nutrition is one of the most important factors that affect animal performance, and it therefore also impacts on financial results in beef systems. In this way, finding the best strategy for feeding supplements is of paramount importance. Aiming to evaluate the effect of supplement feeding strategies for beef cows in the last third of gestation, two experiments were conducted. In Experiment 1, 35 pregnant Nellore cows were assigned to a completely randomized design with four treatments: control, which received no supplement; supplementation for the last 30 d of gestation (30-d; 3.0 kg/d); supplementation for the last 60 d of gestation (60-d; 1.5 kg/d); or supplementation for the last 90 d of gestation (90-d; 1.0 kg/d). All supplemented treatments received the same total amount of supplement throughout the experiment: 90 kg (20% of crude protein). A second experiment (Experiment 2) was delineated to evaluate the effects of the amounts offered in Experiment 1 on intake and metabolism. Four multiparous pregnant Nellore cows were assigned to a 4 × 4 Latin square design, with periods of 15 d each.

**Results:**

There was a linear effect of the number of days of supplementation on calving body weight (BW; *P* < 0.05) and a quadratic effect on BW change from parturition to d 31 post-calving (*P* < 0.05), with cows on the 60-d strategy losing less BW post-calving. No difference was found in offspring birth BW (*P* > 0.10). A significant linear effect on interval from parturition to conception (*P* < 0.05) was observed, with the highest calving to conception interval being observed in the 90-d strategy. The level of supplementation did not affect forage intake or neutral detergent fiber digestibility (*P* > 0.10). Nitrogen excreted through urine tended to increase linearly with the level of supplementation (*P* < 0.10).

**Conclusion:**

Providing 1.5 kg of supplement during the last 60 d of gestation improves cow performance after calving, reducing the magnitude of BW lost, and reduces the number of days from calving to re-conception in the following breeding season compared to the usually recommended period of supplementation of 90 d pre-partum.

## Background

It is estimated that 50% of the cows in extensive beef systems do not receive adequate nutritional management, and this is a main reason for low fertility rates in tropical herds [1].

Among the factors affecting the reproductive performance of beef cattle, nutrition has perhaps the highest impact [2]. Supplementation to grazing animals is a practice that can be adopted under tropical conditions to increase animal performance. Because the last third of gestation usually coincides with the dry season and, consequently, with low quantity and quality of forage, producers in tropical conditions, as in Brazil, are usually oriented to supplement pregnant cows for the last 90 d of gestation.

The objective with this experiment was to study whether this period of supplementation could be reduced to avoid extra labor and, consequently, reduce feeding costs. Therefore, we conducted a study to evaluate the effects of different supplementation strategies for pregnant beef cows in the last third of gestation, receiving supplementation for 90, 60 or 30 d pre-partum.

## Methods

The two experiments were conducted at the Department of Animal Science – Universidade Federal de Viçosa, Brazil, from July to December 2012. All animal care and handling procedures were ethically standardized and approved by the Animal Care and Use Committee of the Universidade Federal de Viçosa, Brazil.

### Experiment 1 – Performance

Thirty-five multiparous, at an average age of five years old, single pregnant Nellore cows with 491.88 ± 55 kg of body weight (BW), a body condition score (BCS) of 4.7 ± 0.58 and 200 ± 15 d of gestation were used. Cows were housed in an experimental area of *Brachiaria decumbens* divided into four paddocks of 5.0 ha each, and had unlimited access to water, feeders and mineral salt (8.7% calcium, 9.0% phosphorous, 18.7% sodium, 9.0% sulfur, 2,400 mg/kg of zinc, 800 mg/kg of copper, 1,600 mg/kg of manganese, 40.0 mg/kg of iodine, 8.00 mg/kg of cobalt and 8.16 mg/kg of selenium). To avoid any effects of paddock on the responses, treatments were rotated among paddocks every 10 d.

Four strategies were evaluated: 30-d – cows received 3.0 kg/d of supplement beginning 30 d prior to calving; 60-d – cows received 1.5 kg/d of supplement beginning 60 d prior to calving; 90-d – cows received 1.0 kg/d of supplement beginning 90 d prior to calving; and control – no concentrate supplement was fed. All supplemented treatments received the same total amount of supplement throughout the experiment, 90 kg/d per head.

Animals were assigned to a completely randomized design with four treatments. There were nine cows each in the control group, 60-d and 90-d supplemented groups, and eight cows in the 30-d supplemented group. Supplement was fed in a collective feeder, which is experimental handling closer to what is normally observed in beef production systems due to cattle gregarious behavior. As the evaluations in Experiment 1 were focused on individual performance, and these measurements were collected individually, the animal was considered the experimental unit, as recommended by Detmann et al. [3].

Supplement was composed of corn, sorghum and soybean meal, and formulated to contain approximately 20% crude protein (CP; Table 1). The level of supplementation adopted for the 90-d treatment corresponds to the daily supply of 1 kg of supplement to approximately 23% of the requirements of CP and 17% of the daily energy requirements of a pregnant Nellore cow, with average BW of 465 kg and expected calf birth BW of 32 kg [4]. The other supplemented treatments were based on the supply of the same total amount of energy and CP, but offered in a reduced period of feeding.

**Table 1:** Ingredients and chemical composition of supplements

Item^a^	Supplement
Ingredients, % as-fed basis	
Corn	33
Sorghum	33
Soybean meal	34
Chemical composition, g/kg	
OM	971
CP	208
apNDF	164

^a^OM – organic matter; CP – crude protein; apNDF – neutral detergent fiber corrected for ash and protein residue

Cow BW was recorded at the beginning of the experiment, approximately 90 d prior to the day of calving, at the week before the expected date of parturition (calving BW), and 31 d after parturition. Cow BCS was recorded at the beginning of experiment, prior to calving and in the first day of the breeding season on a scale ranging from 1 to 9, as recommended by NRC [5] by two experienced technicians. Calf BW was also recorded at birth. Shrunk BW (SBW) was calculated using adjustments proposed by Gionbelli et al. [6] for Nellore cows as follows:

$$ \mathrm{SBW}=0.8084\times {\mathrm{BW}}^{1.0303} $$

After calving, cows were managed as a single herd until after pregnancy was diagnosed. During this period, all cows grazed the same pasture and received mineral supplement ad libitum.

Twenty-one and 31 d after calving, blood samples were taken from the jugular vein using vacuum tubes with clot accelerator and gel for serum separation (BD Vacuntainer® SST II Plus, São Paulo, Brazil). Immediately after collection, the samples were centrifuged at 3,600×g for 20 min. Then, the serum was frozen at −20 °C and subsequently analyzed for progesterone by the chemiluminescent method using Access Progesterone Reagent Kit (Ref. Number 33550, Beckman Coulter®, Brea, USA) in the Access 2 Immunoassay System (Beckman Coulter Inc., Brea, USA).

Pasture chemical composition (Table 2) was assessed by hand-plucked samples every two weeks. In the middle of every experimental month, a second pasture sample was also collected to estimate forage potentially digestible dry matter (pdDM) as proposed by Detmann et al. [5]. Four subsamples were randomly collected in each plot by cutting it close to the ground using a metal square (0.5 m × 0.5 m). Samples were weighed and oven dried at 60 °C for 72 h. After that, samples were mill grinded to pass through a 2-mm screen for indigestible neutral detergent fiber (iNDF) analysis [7]. A sub portion of 20 g of each sample was grinded to pass through a 1-mm screen for analyses of dry matter (DM), ash, crude protein (CP) and neutral detergent fiber (NDF).

**Table 2:** Potentially digestible forage mass and chemical composition of forage in Experiment 1

Item^a,b^	Experimental month		
	1	2	3
pdDM, kg/hm^2^	4820	4050	3410
OM, g/kg	912	913	922
CP, g/kg	77.4	66.6	65.2
NDIN, % of total N	6.94	7.54	9.18
apNDF, g/kg	618	628	679
iNDF, g/kg	225	251	272

^a^pdDM – potentially digestible forage dry matter; OM – organic matter; CP – crude protein; NDIN – neutral detergent insoluble N; apNDF – neutral detergent fiber corrected for ash and protein residue; iNDF – indigestible neutral detergent fiber

^b^pdDM was estimated for forage sampled in the area delimited by a metal square 0.5 m  × 0.5 m ; chemical composition was evaluated in the hand-plucked forage sample

Samples of forage were analyzed following procedures described by Detmann et al. [7] for DM (index INCT-CA G-003/1), CP (index INCT-CA N-001/1), ash (index INCT-CA M-001/1), NDF corrected for contaminant ash and protein (apNDF; index INCT-CA F-002/1, INCT-CA M-002/1, and INCT-CA N-004/1). The iNDF was evaluated using F57 filter bags (Ankom®) by a 288-h in situ incubation procedure [7].

The potentially digestible dry matter (pdDM) was estimated using the second sample collected in each month, as described previously, using the following eq. [5]:

$$ \mathrm{pdDM}\left(\%;\mathrm{drymatterbasis}\right)=0.98\times \left(100-\mathrm{NDF}\right)+\left(\mathrm{NDF}-\mathrm{iNDF}\right) $$

where: 0.98 is the true digestibility coefficient of intracellular content; NDF is forage content of neutral detergent fiber; and iNDF is forage content of indigestible neutral detergent fiber.

In the breeding season, cows were synchronized using the following protocol: on d 0, an intravaginal device of progesterone release (Tecnopec Primer®, São Paulo, Brazil) was inserted, and an i.m. injection of 2.0 mg of estradiol benzoate (Tecnopec RIC-BE®, São Paulo, Brazil) was performed. On day seven, the intravaginal device was removed and cows received a 2-mL injection of cloprosterol sodium (MSD Saúde Animal Ciosin®, São Paulo, Brazil). Finally, on day eight, cows received 0.5 mL of estradiol cypionate i.m. (Zoetis-Pfizer E.C.P.®, Campinas, Brazil). Fixed time artificial insemination (FTAI) was performed 46–52 h following intravaginal device removal (day nine). Semen from five Nellore sires were randomly assigned to each cow. The protocol was repeated once more in a way that cows that did not conceive were inseminated again 32 d after the first FTAI. Pregnancy diagnosis was determined via trans-rectal ultrasonography 30 d after each FTAI. The number of days from parturition to re-conception was calculated for each cow.

Response variables were analyzed using GLIMMIX in SAS 9.4. Initial BW of cows was used as a covariate for data analysis. Treatments were compared using orthogonal contrasts, contrasts were constructed in order to evaluate the effects of supplementation, and the linear and quadratic effects of days receiving supplementation (30, 60 and 90 d). For the variables which didn’t present supplementation effect but a linear or quadratic effect was significant, a Dunnett’s test was performed to identify whether a supplemented treatment differed from the control. Significance was considered at *P* < 0.05.

### Experiment 2 – Intake and metabolism

In order to evaluate the effects of the amounts of supplement offered daily in Experiment 1 on intake and metabolism, a second experiment was conducted simultaneously. Four multiparous, five years old, single pregnant Nellore cows with 488 ± 22 kg of BW, BCS of 4.7 ± 0.3, and 210 ± 10 d of gestation were assigned to a 4 × 4 Latin square design, with experimental periods of 15 d each.

Cows were individually housed in an experimental area of *Brachiaria decumbens* divided into four paddocks of 0.34 ha each, with free access to water, mineral salt and feeders. Experiment 2 started 15 d later than Experiment 1.

The experimental treatments evaluated were: 3.0 kg – cows received 3.0 kg of supplement daily; 1.5 kg – cows received 1.5 kg of supplement daily; 1.0 kg – cows received 1.0 kg of supplement daily; and 0.0 kg – no concentrate supplement was fed. The supplement used was the same used in Experiment 1 (Table 1).

Pasture chemical composition (Table 3) was assessed by hand-plucked samples, collected on the eighth day of each experimental period. On the same day, a second pasture sample was also collected to estimate forage pdDM. Samples were collected and processed as described in Experiment 1.

**Table 3:** Potentially digestible forage mass and chemical composition of forage in Experiment 2

Item^a,b^	Experimental period			
	1	2	3	4
pdDM, kg/hm^2^	4320	3740	3245	1640
OM, g/kg	925	917	922	938
CP, g/kg	68.1	75.7	59.8	52.3
NDIN, % of total N	10.4	10.4	12.3	10.9
apNDF, g/kg	652	644	712	719
iNDF, g/kg	257	239	312	345

^a^pdDM – potentially digestible forage dry matter; OM – organic matter; CP – crude protein; NDIN – neutral detergent insoluble N; apNDF – neutral detergent fiber corrected for ash and protein residue; iNDF – indigestible neutral detergent fiber

^b^pdDM was estimated for forage sampled in the area delimited by a metal square 0.5 m × 0.5 m; chemical composition was evaluated in the hand-plucked forage sample

After six days of adaptation to treatments in each period, a nine-day intake trial was carried out. To estimate fecal excretion, chromium oxide (Cr_2_O_3_) was used as an external marker in the amount of 15 g per animal. The chromium oxide was packed in paper cartridges and delivered via the esophagus with a metal probe once daily, at 10:00. To estimate DM intake, iNDF was used as an internal marker. Once the intake trial had started, six days were allowed for stabilization of Cr_2_O_3_ excretion; and, after that, fecal samples were collected at 1500 h on the seventh day, at 1100 h on the eighth day, and at 0700 h on the ninth day of the intake trial (13^th^, 14^th^ and 15^th^ d of each experimental period, respectively).

Feces samples were collected directly from the rectum of cows, at amounts of approximately 200 g, dried (60 °C/72 h) and mill grinded as described for forage samples. Ground samples were proportionally combined to a pooled three-day sample per animal per period.

Samples of forage, feces and supplement were analyzed for DM, CP, ash, apNDF, and iNDF following procedures previously described for Experiment 1. Fecal samples were also analyzed for levels of chromium by atomic absorption spectrophotometry (index INCT-CA M-005/1) and titanium dioxide by colorimetry (index INCT-CA M-007/1), as recommended by Detmann et al. [8].

Fecal excretion (FE) was estimated by the ratio of chromium oxide and its concentration in the feces. Dry matter intake (DMI) was estimated by using iNDF as an internal marker and calculated by the following equation:

$$ \mathrm{DMI}\left(\mathrm{kg}/\mathrm{d}\right)=\left[\left(\mathrm{FE}\times \mathrm{iNDFfeces}-\mathrm{iNDFsupplement}\right)\div \mathrm{iNDFforage}\right]+\mathrm{SI} $$

where FE is the fecal excretion (kg/d); iNDF feces is the concentration of iNDF in the feces (kg/kg); iNDF supplement is the iNDF in the supplement (kg/d); iNDF forage is the concentration of iNDF in forage (kg/kg) and SI is DM supplement intake.

At the 15^th^ d of each period, two blood samples were collected immediately before and 4 h after supplementation, to estimate insulin levels pre- and post-supplementation, respectively. Blood was collected in tubes with clot activator and gel for serum separation (BD Vacuntainer® SST II Plus, São Paulo, Brazil), centrifuged at 3,600×g for 20 min and serum was immediately frozen at −20 °C in duplicate until further analysis. The same blood collected 4 h after supplementation was used for quantification of serum urea concentrations.

Insulin was analyzed by the chemiluminescent method using Access Ultrasensitive Insulin Reagent (Ref. Number 33410, Beckman Coulter®, Brea, USA) in the Access 2 Immunoassay System (Beckman Coulter Inc., Brea, USA). Urea was quantified by an enzymatic-colorimetric method using reagents provided by commercial kits (Ref. Number K056, Bioclin® Quibasa, Belo Horizonte, Brazil) in an automatic biochemistry analyzer (Mindray BS200E, Shenzhen, China). Serum urea N (SUN) was estimated as 46.67% of total blood urea.

Spot urine sampling at the 15^th^ day of each experimental period (collected immediately before the 4 h after supplementation blood sampling) was used to assess the excretion of urinary nitrogenous compounds [9]. Urine volume was estimated using creatinine concentration as a marker and assuming a daily creatinine excretion (mg/d) of 34.5 × SBW^0.9491^ [10]. Microbial N synthesis was estimated by using the technique of the purine derivatives in urine. Allantoin was estimated by colorimetry [11]. The urinary concentrations of creatinine and uric acid were obtained by colorimetric and enzymatic-colorimetric methods, respectively. The analyses of creatinine and uric acid were performed in an automatic biochemistry analyzer (Mindray BS200E, Shenzhen, China) using commercial kits (Ref. Number K067 for creatinine and K139 for uric acid, Bioclin® Quibasa, Belo Horizonte, Brazil).

Excretion of the purine derivatives in urine was calculated by the sum of the allantoin and uric acid excretions, which were obtained by the product between their concentrations in urine by the daily urinary volume. Absorbed purines were calculated from the excretion of purine derivatives [12], as follows:

$$ Y\kern0.5em =\kern0.5em {\frac{x-0.301\times BW}{0.8}}^{0.75} $$

where Y = absorbed purines (mmol/d), *x* = excretion of purine derivatives (mmol/d), 0.8 = recovered absorbed purines. The 0.301 × BW^0.75^ value = endogenous excretion of purine derivates.

Ruminal synthesis of nitrogen compounds was calculated as a function of the absorbed purines [12]:

$$ Z=\frac{70\times Y}{0.93\times 0.137\times 1,000} $$

where Z = ruminal synthesis of nitrogen compounds (g/d), Y = absorbed purines (mmol/d), 70 = purine N content (mg/mol), 0.93 = purine digestibility and 0.137 = relation of purine N:total N of microorganisms.

Linear, quadratic and cubic effects of amount of supplement fed daily were analyzed using GLIMMIX in SAS 9.4. Animal and period were considered as random effects. Significance was assumed at *P* < 0.05.

## Results

### Experiment 1

There was a linear effect of number of days of supplementation on cow BW at calving (Table 4; *P* < 0.05). Body weight at calving ranged from 508 kg for cows in the 30-d strategy to 531 kg for cows in the 90-d strategy. Supplemented treatments did not differ from the control by Dunnett’s test as well. Looking at the individual means of each treatment, can be observed that, actually, control cows had a BW at calving intermediate compared to the supplemented cows. It possibly reflects the small numbers of animals used in this study coupled with the variability of the variable in question. Birth BW of calves averaged 34.4 kg and was not different among supplementation strategies used for their mothers in the last third of gestation (*P* > 0.10).

**Table 4:** Cow BW and BCS, calf BW, cow progesterone concentrations and reproductive performance

Item^a^	Treatment^b^				SEM	*P*-value^c^		
	30-d	60-d	90-d	Control		S	L	Q
Supplement fed, kg/d	3.00	1.50	1.00	–				
Cow BW, kg, cow BCS and calf BW, kg								
Initial BW	494	517	503	503	18.0	0.95	0.74	0.43
Initial BCS	4.66	4.65	4.54	4.87	0.20	0.28	0.65	0.85
Calving BW	508	515	531	522	6.79	0.56	0.02	0.55
Calving BCS	4.74	4.83	4.83	4.82	0.15	0.95	0.70	0.82
Calf birth BW	33.8	31.7	35.8	36.2	1.93	0.28	0.43	0.10
Cow BW 31 d after calving	468	490	482	465	7.73	0.09	0.21	0.15
BW change from parturition to day 31 post-calving	−46.7	−24.9	−53.5	−62.1	8.24	0.04	0.58	0.03
Breeding season BCS	5.00	5.11	4.83	5.03	0.21	0.84	0.57	0.43
Progesterone concentration, ng/dL								
21 d after calving	0.27*	0.21	0.12	0.14	0.06	0.34	0.05	0.82
31 d after calving	1.24	0.70	0.69	0.14	0.37	0.08	0.29	0.55
Cow reproductive performance, %								
Calving to conception, d	57.2	63.1	84.2*	64.1	5.85	0.53	0.01	0.30

^a^BW– body weight; BCS – body condition score; FTAI – fixed time artificial insemination

^b^Treatments: 30-d – cows received 3.0 kg of concentrate supplement beginning 30 d prior to calving; 60-d – cows received 1.5 kg of concentrate supplement beginning 60 d prior to calving; 90-d – cows received 1.0 kg of concentrate supplement beginning 90 d prior to calving; and control – no concentrate supplement was fed

^c^S – effect of supplementation, supplemented treatments compared to the control; L and Q – effects of linear and quadratic order of supplement delivery strategy (30, 60 or 90 d). * Means statistically different from the control by Dunnett’s test

Thirty-one days after calving, cows that received supplementation pre-calving tended to be heavier (480 kg, *P* < 0.10) compared to control cows (465 kg). Supplemented cows also lost less BW from parturition to 31 d post-calving (*P* < 0.05); within the supplemented treatments, there was a quadratic effect of the number of days of supplementation on post-partum BW change (*P* < 0.05): cows from the 60-d strategy lost less BW (−25 kg) compared to cows in the 30-d and 90-d strategies (−50 kg on average). Nevertheless, supplementation or level of supplementation did not affect BCS at any time (*P* > 0.10).

At 21 d post-calving, progesterone concentration linearly reduced with an increase in days of supplementation (*P* = 0.05), cows in the 30-d strategy had higher progesterone concentration compared to control cows. Ten days later, at 31 d post-calving, supplemented cows tended to have greater progesterone concentration compared to cows receiving no supplement (*P* < 0.10).

A significant linear effect on interval from parturition to conception (*P* < 0.05) was observed, with the highest calving to conception interval being observed for cows in the 90-d strategy.

### Experiment 2

Level of supplementation linearly increased DM intake (kg/d and g/kg of BW), DM digested (kg/d and g/kg of BW), OM (kg/d and g/kg of BW) and OM digested (kg/d), and crude protein (g/d – Table 5; *P* < 0.05). There was also a cubic effect of the level of supplementation on intake of crude protein (g/d; *P* < 0.05). There was no effect of the level of supplementation on intake (g/d and g/kg BW) of forage DM, forage OM, forage apNDF, apNDF and iNDF.

**Table 5:** Intake according to amount of supplement fed to cows in the last third of gestation

Item^a^	Treatment^b^				SEM	*P*-value^c^		
	3.0 kg	1.5 kg	1.0 kg	0.0 kg		L	Q	C
Intake per day, kg/d								
Forage DM	5.56	5.69	6.00	5.61	0.72	0.96	0.73	0.86
DM	8.03	6.92	6.82	5.61	0.72	0.05	0.94	0.15
DM digested	4.00	2.97	2.76	2.08	0.45	0.01	0.67	0.08
Forage OM	5.11	5.26	5.55	5.22	0.65	0.90	0.72	0.89
OM	7.51	6.46	6.35	5.22	0.65	0.05	0.95	0.15
OM digested	4.03	3.01	2.83	2.13	0.41	0.01	0.68	0.06
Forage apNDF	3.70	3.89	4.11	3.85	0.48	0.83	0.66	0.94
apNDF	4.11	4.09	4.25	3.85	0.48	0.72	0.71	0.68
iNDF	1.56	1.69	1.71	1.60	0.19	0.88	0.53	0.95
CP, g/d	882	613	556	359	58.8	<0.01	0.48	<0.01
Intake per kg BW, g/kg BW								
Forage DM	9.95	9.78	10.15	9.58	1.22	0.84	0.88	0.77
DM	14.35	11.92	11.53	9.58	1.23	0.03	0.85	0.12
DM digested	7.16	5.09	4.68	3.55	0.77	0.01	0.54	0.07
Forage OM	9.14	9.05	9.39	8.92	1.12	0.89	0.87	0.81
OM	13.42	11.13	10.74	8.92	1.13	0.03	0.84	0.12
OM digested	5.06	4.74	4.75	6.25	0.95	0.24	0.20	0.39
Forage apNDF	6.64	6.70	6.94	6.59	0.84	0.97	0.81	0.87
apNDF	7.36	7.05	7.17	6.59	0.84	0.54	0.88	0.61
iNDF	2.81	2.93	2.87	2.74	0.33	0.89	0.70	0.99

^a^DM – dry matter; OM – organic matter; apNDF – neutral detergent fiber corrected for ash and protein residue; iNDF – indigestible neutral detergent fiber; CP – crude protein

^b^Treatments: 3.0 kg – cows received 3.0 kg of concentrate daily; 1.5 kg – cows received 1.5 kg of concentrate daily; 1.0 kg – cows received 1.0 kg of concentrate daily; and 0.0 kg – no concentrate supplement was fed

^c^L, Q and C – effects of linear, quadratic and cubic order of level of supplementation

Level of supplementation increased the digestibility of OM and CP linearly (*P* < 0.05; Table 6). There was also a cubic effect of the level of supplementation on digestibility of OM (*P* < 0.05).

**Table 6:** Coefficients of digestibility (%) according to amount of supplement fed

Item^a^	Treatment^b^				SEM	*P*-value^c^		
	3.0 kg	1.5 kg	1.0 kg	0.0 kg		L	Q	C
OM	53.87	45.94	44.44	39.57	3.30	<0.01	0.42	0.01
apNDF	52.39	49.52	50.22	48.77	2.55	0.18	0.69	0.25
CP	48.12	39.14	36.54	17.70	8.98	0.03	0.53	0.11

^a^DM – dry matter; OM – organic matter; apNDF – neutral detergent fiber corrected for ash and protein residue; CP – crude protein

^b^Treatments: 3.0 kg – cows received 3.0 kg of concentrate daily; 1.5 kg – cows received 1.5 kg of concentrate daily; 1.0 kg – cows received 1.0 kg of concentrate daily; and 0.0 kg – no concentrate supplement was fed

^c^L, Q and C – effects of linear, quadratic and cubic order of level of supplementation

The microbial N produced (g/d), the efficiency of microbial N produced in relation to N ingested and the efficiency of microbial N produced in relation to OM digested were not different among levels of supplementation (*P* > 0.10; Table 7). Level of SUN was also similar among treatments (*P* > 0.10), but ureic nitrogen excreted (g/d, UUN) tended to increase linearly with level of supplementation (*P* < 0.10).

**Table 7:** Nitrogen utilization according to amount of supplement fed

Item^a^	Treatment^b^				SEM	*P*-value^c^		
	3.0 kg	1.5 kg	1.0 kg	0.0 kg		L	Q	C
Nmic, g/d	72.87	77.99	48.02	36.14	16.4	0.17	0.58	0.82
Nmic, g/g N ingested	0.747	0.788	0.605	0.533	0.20	0.45	0.76	0.93
Nmic, g/kg OMD	26.71	26.21	19.99	14.87	7.19	0.25	0.72	0.67
SUN, mg/dL	13.77	12.72	12.37	10.85	1.70	0.20	0.88	0.41
UUN, g/d	61.36	47.59	33.35	32.39	9.85	0.09	0.53	0.48

^a^Nmic – microbial N; OMD – organic matter digested; SUN – Serum urea nitrogen; UUN – Urine urea N

^b^Treatments: 3.0 kg – cows received 3.0 kg of concentrate daily; 1.5 kg – cows received 1.5 kg of concentrate daily; 1.0 kg – cows received 1.0 kg of concentrate daily; and 0.0 kg – no concentrate supplement was fed

^c^L, Q and C – effects of linear, quadratic and cubic order of level of supplementation

Pre-supplementation levels of insulin were not different according to the level of supplementation (*P* > 0.10; Table 8), but insulin levels 4 h after supplementation increased linearly with the amount of supplement fed (*P* < 0.05).

**Table 8:** Insulin levels (μIU/mL) according to amount of supplement fed

Item	Treatment^a^				SEM	*P*-value^b^		
	3.0 kg	1.5 kg	1.0 kg	0.0 kg		L	Q	C
Pre-supplementation	1.68	1.50	1.18	1.13	0.43	0.13	0.79	0.63
Post-supplementation	2.20	1.48	1.33	1.25	0.38	0.03	0.21	0.13

^a^Treatments: 3.0 kg – cows received 3.0 kg of concentrate daily; 1.5 kg – cows received 1.5 kg of concentrate daily; 1.0 kg – cows received 1.0 kg of concentrate daily; and 0.0 kg – no concentrate supplement was fed

^b^L, Q and C – effects of linear, quadratic and cubic order of level of supplementation

## Discussion

Nutritional status at calving is the most important factor that influences the interval from parturition to conception in beef cows. Postpartum nutrient intake can modulate the duration of the postpartum anestrous interval; however, if thin cows gain great amounts of weight after calving, ovulation occurs later than for cows that calve in good body condition and maintain body weight [2].

Cabral et al. [13] supplemented pregnant cows grazing pastures in similar conditions to the present study, but with different amounts of supplement per day, and observed a quadratic pattern in performance, with cows receiving 1.0 kg of supplement daily gaining more BW. The total amount of supplement provided for the 1.0 kg treatment in Cabral’s work (84 kg) was similar to the present study (90 kg).

Based on the magnitude of post-partum BW change, supplementing cows with 1.5 kg of supplement during the last 60 d prior to calving was the most efficient strategy to lessen the post-partum negative energy balance, since cows on this strategy lost about half (−25 kg) of the BW lost in the 30-d and 90-d strategies (−50 kg). The differences found in BW changes are related to supplementing proper amounts of nutrients at key times and the effects when this supplement is removed from cow diet and its impact on cow metabolism, in addition to changes that naturally occur due to calving and lactation. Therefore, supplementing adequate amounts of nutrients at times of higher requirements (last 60 d of gestation) [4, 5] seems to metabolically prepare the cow for the post-calving period, not only due to accretion in body reserves, but also due to its effects on cow metabolism later on, when supplements are no longer provided. These results provide evidence that adopting a strategy to deliver the same amount of supplement 60 d prior to calving, instead of the usually recommended 90 d, may not only be economical, but also improve cow post-partum performance.

Previous studies in cattle during late gestation [14] have provided evidence that feeding systems during the last third of gestation can alter the subsequent birth weight of the progeny, suggesting that the maternal dietary energy source may affect fetal growth [15]. Although the maternal intake of protein has also been shown to be an important factor for fetal growth, Summers et al. [16] found no difference in calf birth BW according to the supplementation strategy applied to their mothers. Similarly, no difference was observed for calf birth BW in the present study, probably because throughout the experiment forage presented median quality (Table 2).

In tropical pastures, the use of energetic-protein supplements can impact forage intake in different ways, depending largely on forage quality, amount and quality of supplement provided [17–19]. Linear increase of DM, OM and CP intake with levels of supplementation observed in the present work was simply due to the increase in supplement intake, as no difference in forage DM intake was observed in the present study.

During the intake and metabolism experiment (Table 3), average forage CP content was adequate or slightly below the minimum required by ruminal microorganisms [17]. In this way, the level of supplementation did not affect apNDF digestibility or intake.

Level of supplementation linearly increased insulin levels post-supplementation. For animals in an exclusive forage diet, the relative amount of propionate, a glycogenic precursor, available for metabolism is low, and supplementation significantly increases the proportion of propionate produced in the rumen [20].

Hawkins et al. [21] have suggested that an increase in insulin, concomitant with a decrease in growth hormone (GH), is an important relationship to consider for evaluating the impact of nutrition on reproduction. Insulin is an important mediator of nutritional effects on follicular dynamics in cattle, and can stimulate the release of gonadotropin-releasing hormone (GnRH) from the hypothalamus. In the ovaries, insulin may also stimulate cell proliferation and steroidogenesis [22]. In accordance with these findings, in the present study, supplementing with higher amounts of supplement daily linearly increased progesterone concentrations after calving.

The interval from calving to conception greatly influences the profitability of beef production. Hence, in beef systems, it is recommended that the calving interval is no longer than 85 d in order to assure the cow will produce a calf per year. Cows that conceive early calve early, and have a better opportunity to start reproductive cycles in time to re-conceive in the next breeding season. The calving date also affects the value of offspring, demonstrating the importance of supplementation strategies to improve early re-conception.

Cushman et al. [23] and Funston et al. [24] reported that heifer calves born early tend to conceive early in their first breeding season and remain in the herd. The calving date can also impact male offspring performance; steer calves born earlier in the calving season have greater weaning BW, hot carcass weight and marbling scores [24]. In this way, increasing early calving by early conception may increase progeny value at weaning, enhance carcass value of the steers and increase heifer pregnancy rates in their first breeding season.

All treatments in the present study presented acceptable calving intervals, but cows receiving higher amounts of supplement per day for a reduced number of days had lower calving intervals (30-d and 60-d vs. 90-d).

## Conclusions

Providing 1.5 kg of supplement during the last 60 d of gestation, instead of 90 d that are usually recommended, is a nutritional management strategy that can be adopted to improve cow performance, reducing the magnitude of BW lost after calving and reducing the number of days from calving to re-conception in the following breeding season, with no negative effect on forage intake or digestibility.

## Abbreviations

apNDF: Neutral detergent fiber corrected for ash and protein residue
BCS: Body condition score
BW: Body weight
CP: Crude protein
DM: Dry matter
FTAI: Fixed time artificial insemination
iNDF: Indigestible neutral detergent fiber
NDF: Neutral detergent fiber
OM: Organic matter
pdDM: potentially digestible dry matter
SUN: Serum urea N
UUN: Urine urea N
